# The HTLV-1 oncoprotein Tax is modified by the ubiquitin related modifier 1 (Urm1)

**DOI:** 10.1186/s12977-018-0415-4

**Published:** 2018-04-17

**Authors:** Rita Hleihel, Behzad Khoshnood, Ingrid Dacklin, Hayssam Omran, Carine Mouawad, Zeina Dassouki, Marwan El-Sabban, Margret Shirinian, Caroline Grabbe, Ali Bazarbachi

**Affiliations:** 1 Department of Internal Medicine, Faculty of Medicine, American University of Beirut, Beirut, Lebanon; 2 Department of Anatomy, Cell Biology and Physiological Sciences, Faculty of Medicine, American University of Beirut, Medical Center, P.O. Box 113-6044, Beirut, Lebanon; 3 Department of Molecular Biology, Umeå University, Building 6L, 901 87 Umeå, Sweden; 4 Department of Experimental Pathology, Immunology and Microbiology, Faculty of Medicine, American University of Beirut, Beirut, Lebanon; 5 Department of Biology, Faculty of Sciences 3, Lebanese University, Tripoli, Lebanon

**Keywords:** ATL, HTLV-1, Tax, Urm1, NF-κB, Oncogenesis

## Abstract

**Background:**

Adult T-cell leukemia/lymphoma (ATL) is an aggressive malignancy secondary to chronic human T-cell lymphotropic virus 1 infection, triggered by the virally encoded oncoprotein Tax. The transforming activity and subcellular localization of Tax is strongly influenced by posttranslational modifications, among which ubiquitylation and SUMOylation have been identified as key regulators of the nuclear/cytoplasmic shuttling of Tax, as well as its ability to activate NF-κB signaling.

**Results:**

Adding to the complex posttranslational modification landscape of Tax, we here demonstrate that Tax also interacts with the ubiquitin-related modifier 1 (Urm1). Conjugation of Urm1 to Tax results in a redistribution of Tax to the cytoplasm and major increase in the transcription of the NF-ĸB targets *Rantes* and *interleukin*-*6*. Utilizing a *tax*-transgenic *Drosophila* model, we show that the Urm1-dependent subcellular targeting of Tax is evolutionary conserved, and that the presence of Urm1 is strongly correlated with the transcriptional output of Diptericin, an antimicrobial peptide and established downstream target of NF-κB in flies.

**Conclusions:**

These data put forward Urm1 as a novel Tax modifier that modulates its oncogenic activity and hence represents a potential novel target for developing new strategies for treating ATL.

## Background

The Human T-cell Leukemia Virus type 1 (HTLV-1) transactivator Tax initiates adult T-cell leukemia/lymphoma (ATL), an aggressive T-cell lymphoproliferative malignancy with poor prognosis [1, 2]. Through multiple cellular effects such as activation of NF-κB signalling, inhibition of apoptosis and interfering with DNA repair, Tax triggers an oncogenic phenotype and often confers resistance to chemotherapy [3–6]. By inducing proteasomal degradation of Tax, the combination of arsenic trioxide (arsenic) and interferon-alpha (IFN) selectively kills ATL cells and has the potential to cure ATL in mice and patients [7–13].

Tax is post-translationally modified by phosphorylation, acetylation and O-GlcNAcylation, as well as the attachment of ubiquitin-like molecules (UBLs), such as ubiquitin and the small ubiquitin modifiers (SUMOs) [14–19]. Ubiquitylation and SUMOylation have been reported to regulate the activity, turnover, subcellular localization, and protein-protein interactions of Tax [8, 20–23].

Ubiquitin-related modifier 1 (Urm1) is a dual function UBL [24, 25] with an established role as a sulfur transferase important for tRNA modification [26–29], as well as a posttranslational modifier that conjugates to target proteins during oxidative stress [30, 31]. An evolutionary conserved role of Urm1 for survival and oxidative stress responses has recently been reinforced by our recent report on the Urm1/Uba4 conjugation machinery in *Drosophila melanogaster* [32]. In this study we have unravelled Tax as a novel target of urmylation in both humans and flies and demonstrated a patho-physiologically relevant role of Urm1 as a regulator of the subcellular localization, as well as signaling activity of Tax.

## Results

### The HTLV-1 oncoprotein Tax is urmylated and subsequently localized to the cytoplasm

We have previously showed that the combination of arsenic and IFN induces G1 arrest and apoptosis in ATL leukemic cells, associated with induction of oxidative stress and Tax degradation in the proteasome [7, 8, 10, 12]. Importantly, degradation of Tax degradation is mediated by PML-dependent SUMOylation, followed by RNF4 dependent ubiquitylation [8]. Given that Tax is heavily modified by ubiquitin and SUMO (SUMO-1, 2 and 3), we were interested to analyze whether it is also modified by another ubiquitin-like molecule, Urm1, which is known to rapidly conjugate to multiple target proteins in response to oxidative stress. Indeed, arsenic is known to induce oxidative stress [33] and the combination of arsenic and IFN results in Tax recruitment to PML nuclear bodies, a well-known stress sensor [7–12].

**Fig. 1:**
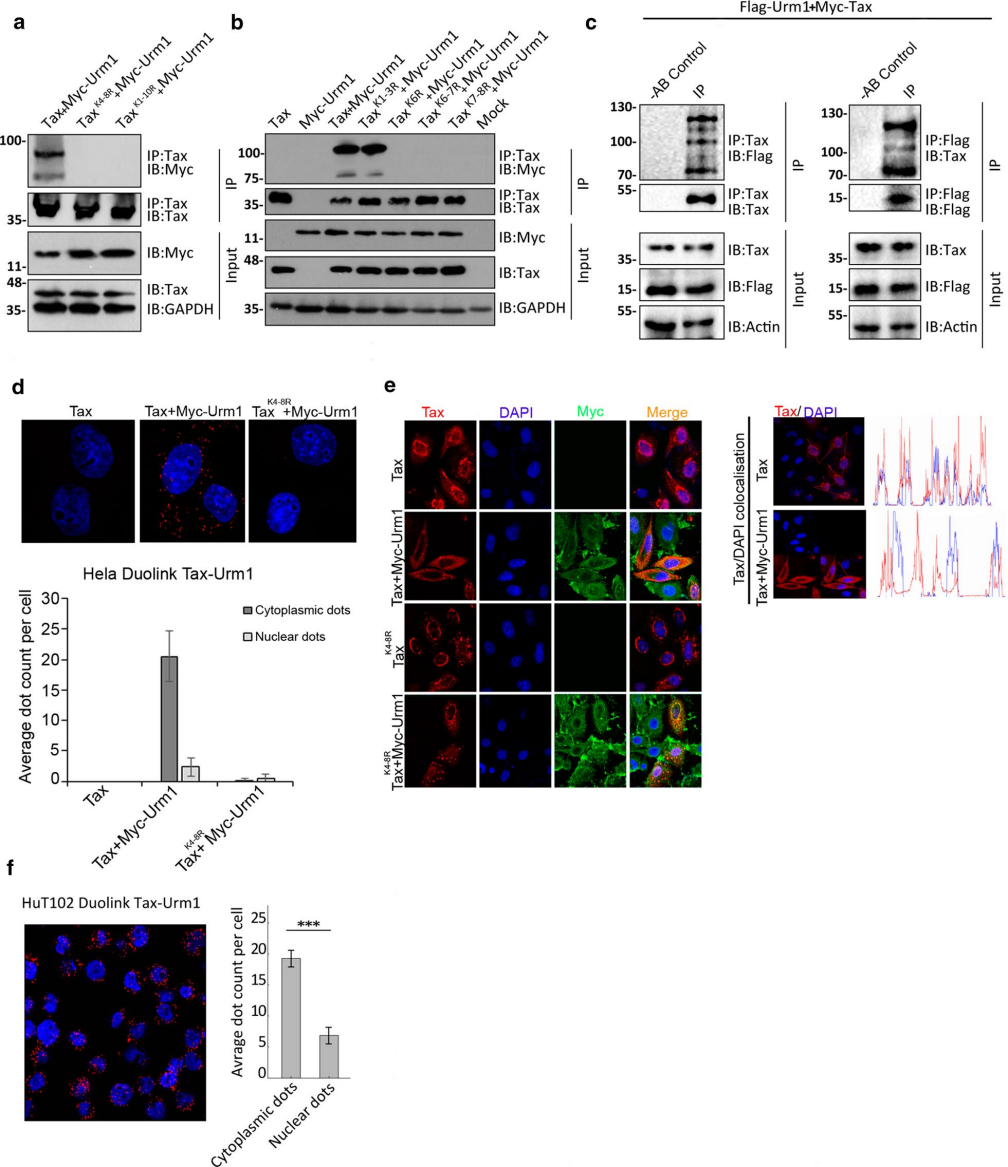
Urmylation of the HTLV-1 oncoprotein Tax strongly influences the subcellular localization of Tax. **a** Tax is targeted by urmylation upon co-expression of wild-type Tax and Myc-tagged Urm1 in HeLa cells. Mutagenesis of Tax, replacing all lysine residues (Tax^K1-10R^) or lysine 4-8 (Tax^K4-8R^) for arginines, completely abolished Urm1 conjugation to Tax. **b** Mutational analysis of the indicated lysine residues in Tax, expressed in HeLa cells, indicates that Urm1 targets the same lysine residues as ubiquitin and SUMO for conjugation, lysine residues 4-8. **c** Tax is urmylated in protein lysates derived from *Drosophila melanogaster* adult eyes expressing Myc-Tax and Flag-Urm1 under control of the GAL4/UAS system, using GMR-GAL4 as driver. – AB control represents a negative control, in which no antibody was added to the immunoprecipitation. **d** Duolink^®^ in situ proximity ligation assay performed in HeLa cells expressing Tax and Myc-tagged Urm1, depicting that Tax-Urm1 protein complexes are primarily localized in the cytoplasmic compartment *n* = 25, *P* < 0.0001. **e** Co-expression of Myc-Urm1 (green) and Tax (red) in HeLa cells causes a shift in the subcellular localization of Tax, with a clear increase of cytoplasmic Tax, as compared to cells expressing Tax alone (two upper panels). The Urm1-dependent nuclear exclusion of Tax is abrogated in cells expressing the Tax^K4-8R^ mutant, indicating that lysine 4-8 is required for Urm1-mediated regulation of Tax (two lower panels). **f** Also in ATL-derived HuT102 cells, interaction between endogenous Tax and endogenous Urm1 proteins is primarily encountered in the cytoplasm, *n* = 42, *P* < 0.0001. Representative images for the Duolink^®^ experiments and immunohistochemical analysis in HeLa cells were acquired by confocal microscopy using either a Zeiss LSM 510 META confocal laser microscope or a Zeiss LSM 710 confocal microscope (Zeiss, Oberkochen, Germany) with a Plan Apochromat 63/1.4 numeric aperture oil-immersion objective using Zen 2009 (Carl Zeiss). High-resolution images were obtained with a deconvolution program (Autodeblur; Image Quant), using blind iterative algorithms

Initially, we used *tax*-transfected HeLa cells to confirm that Tax is modified by urmylation, and found Urm1 to conjugate to wild type Tax, but not to a Tax mutant where all lysine residues were replaced by arginine, TaxK1-10, or a Tax variant in which only lysines 4-8 were mutated, TaxK4-8R (Fig. 1a). In an attempt to further map the lysine targeted by Urm1, we concluded that most likely multiple Urm1 moieties attach to Tax via lysine residues 4-8, since neither of the tested mutants in this area could be urmylated in our assay (Fig. 1a, b). This is interesting in light of the reported targeting of lysine 4-8 by both ubiquitin and SUMO [16, 17]. In addition, utilizing an established transgenic *Drosophila* model for Tax-driven transformation [32], in which Myc-tagged Tax is expressed under control of the UAS/GAL4 system, we further confirmed that Tax is urmylated also when expressed in fly tissues, and that similar to HeLa cells, the conjugation of Urm1 to Tax results in a size shift of Tax also in fly lysates (Fig. 1c). To decipher the intracellular localization of Tax/Urm-1 interaction, we utilized the proximity ligation assay (Duolink^®^) methodology. We found that Urm1 interacts with Tax in the cytoplasm (Fig. 1d). Since modification by other UBLs strongly influences the subcellular localization of Tax, we tested the impact of Urm1 overexpression on the subcellular localization of Tax (Fig. 1e). Immunofluorescence analysis demonstrates that whereas transfected Tax has a predominant nuclear distribution, a significant shift to the cytoplasm is observed upon addition of URM-1 (Fig. 1f). In agreement with a targeting of lysines 4-8 by Urm1, TaxK4-8R mutant failed to interact with Urm1 in these cells (Fig. 1d) and showed a similar subcellular localization both in the presence and absence of Urm1 (Fig. 1e), implying that the localization of TaxK4-8R mutant is not affected by Urm1. Importantly, in ATL-derived HuT-102 cells, cytoplasmic interaction of endogenous Tax and endogenous Urm1 was also evident (Fig. 1f).

**Fig. 2:**
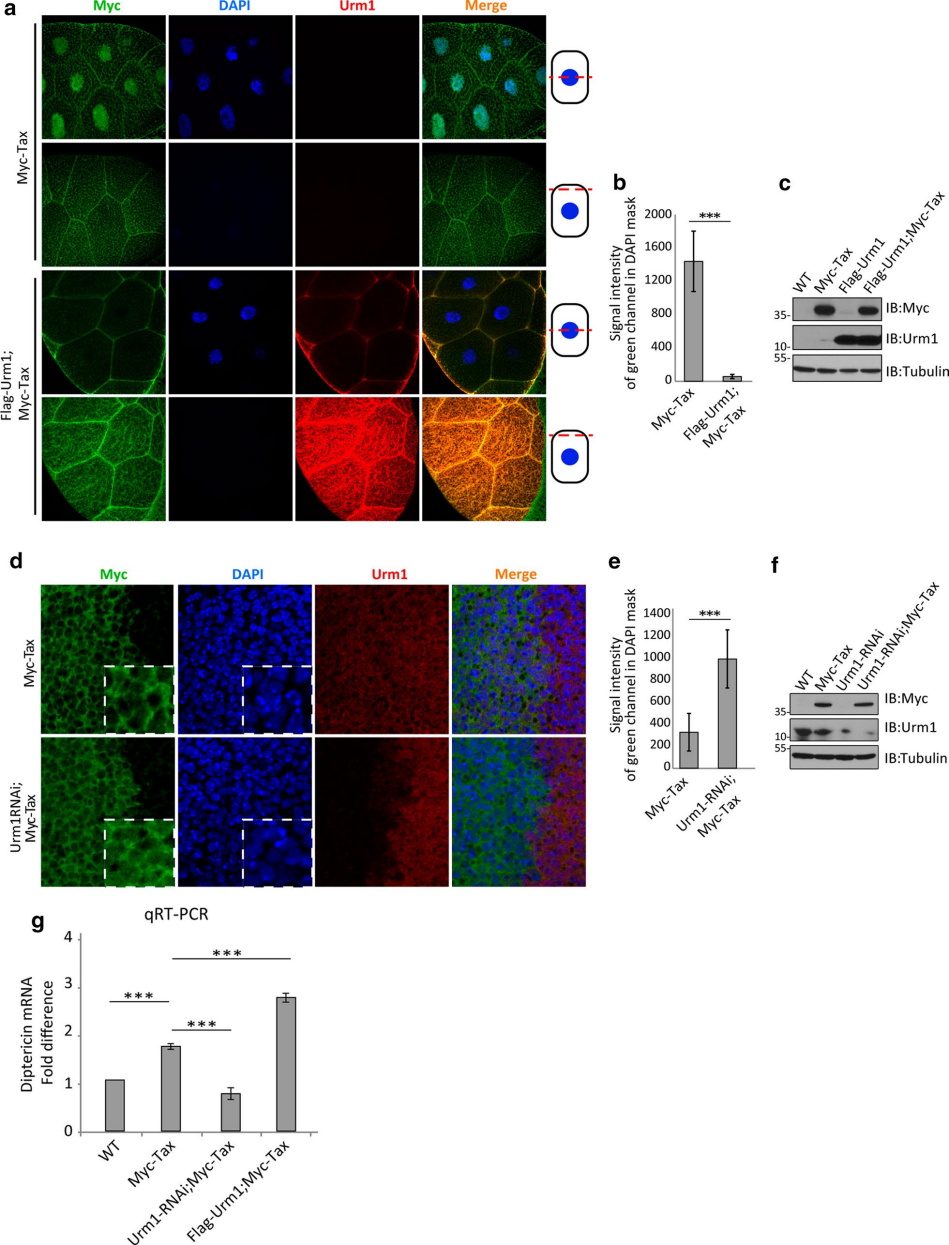
Posttranslational modification of Tax by urmylation regulates the nuclear-cytoplasmic shuttling of Tax, and in extension its ability to activate NF-κB signaling. **a** Co-expression of Flag-Urm1 (red) and Myc-Tax (green) in 3rd instar larval *Drosophila* salivary glands (employing Sgs-GAL4 as driver) results in a complete blockage of the nuclear Myc-Tax accumulation, which is observed upon expression of Myc-Tax alone. Quantification is shown in (**b**), *n* = 12, *P* < 0.0001. **c** Verification of the expression levels of Myc-Tax and Flag-Urm1, induced by the UAS/GAL4 system in *Drosophila*, by Western Blot. **d** RNAi-mediated knockdown of Urm1 promotes a significant increase in the amount of nuclear Tax, analyzed in the 3rd instar larval wing disc of flies with the indicated genotypes. Engrailed-GAL4 was used to drive expression of Myc-Tax (green) alone (top) and together with Urm1-RNAi (red) (bottom) specifically in the posterior half of the wing disc (left side of each image), while preserving wild-type tissue in the anterior part (right side of each image). The images are taken at the border between the anterior and posterior side, thus displaying the control wild-type tissue as well as the genetically modified area expressing Myc-Tax and/or Urm1-RNAi in the same view. Quantification of Tax/DAPI colocalization is shown in (**e**), *n* = 14, *P* < 0.0001. **f** Verification of the GAL4/UAS-mediated induction of Myc-Tax expression and the efficiency of RNAi-mediated knockdown of Urm1 in *Drosophila*, visualized by Western Blot. **g** The ability of Tax to induce activation of the NF-κB pathway is strongly correlated with the expression levels of Urm1, as indicated by qRT-PCR analysis of Diptericin, an established transcriptional target of NF-κB in the adult *Drosophila* fat body. Expression of Myc-Tax, Urm1-RNAi and Flag-Urm1 was induced by the UAS/GAL4 system, utilizing FB-GAL4 as driver, and the qRT-PCR was performed in triplicates on two biological replicates (*P* < 0.001). Images depicting *Drosophila* salivary glands in (**a**) and wing imaginal discs in (**d**) were acquired using a Nikon C1 confocal microscope, magnifications, ×60 Plan Apo VC NA 1,40 oil and ×100 Plan Apo VC NA 1,40 oil and EZ-C1 software. The Duolink^®^ images were acquired as described in Fig. 1

Next, we investigated whether the Urm1-mediated shuttling of Tax is conserved also in *Drosophila*. In third instar larval salivary glands, which commonly are used to visualize nuclear-cytoplasmic shuttling of molecules, co-expression of Urm1 and Tax by the UAS/GAL4-system caused a marked export of Tax out from the nucleus (Fig. 2a–c). In agreement with an Urm1-dependent cytoplasmic targeting of Tax, RNAi-mediated reduction of Urm1 protein levels reciprocally resulted in increased levels of nuclear Tax, as compared to expressing Tax alone (Fig. 2d–f).

### Nuclear-cytoplasmic shuttling of Tax is modulated upon urmylation *in vivo* and *in vitro*

Tax-related oncogenesis has been linked to the activation of several pro-survival and proliferative signaling pathways, among which NF-ĸB is most prominent. To investigate whether urmylation affects the signaling activity of Tax, we monitored the mRNA levels of the antimicrobial peptide Diptericin, an established downstream transcriptional target of NF-ĸB in *Drosophila*, as readout for NF-ĸB activity. Since the primary source of Diptericin in *Drosophila* is the fat body, a fat-body specific GAL4 driver (FB-GAL4) was used to assess the signaling capacity of Tax in the presence and absence of Urm1. As expected, we found a clear correlation between Urm1-dependent nuclear/cytoplasmic shuttling of Tax and Diptericin transcription, as indicated by a threefold induction of Diptericin in flies co-expressing Tax and Urm1, and a concomitant reduction in flies with reduced levels of Urm1 (Fig. 2g).

### Urm1 strongly influence Tax-induced transcription of NF-ĸB target genes

**Fig. 3:**
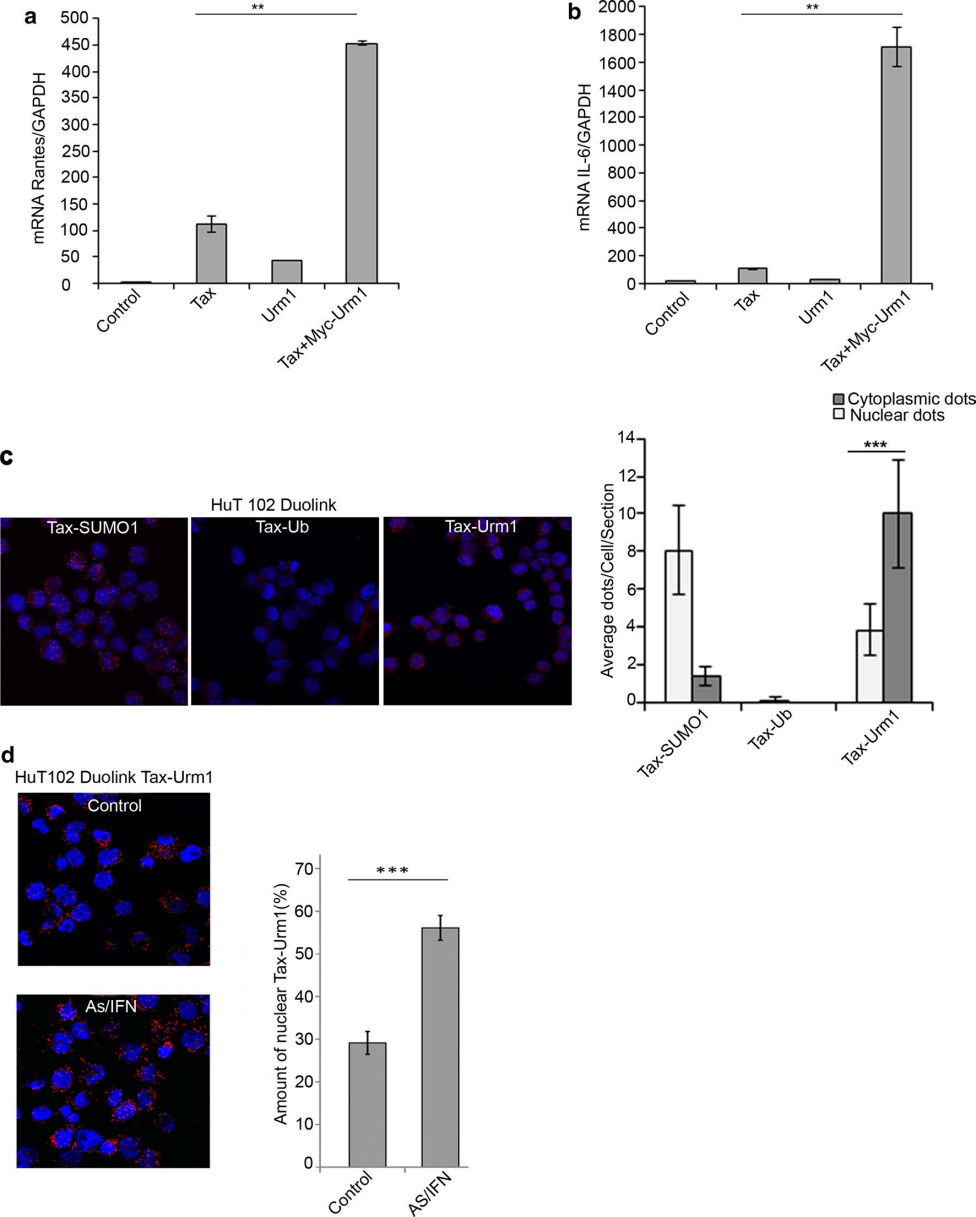
Urm1 augments Tax-induced transcriptional upregulation of NF-ĸB target genes. **a**, **b** The mRNA levels of *Rantes* and *IL*-*6*, both known as transcriptional targets of NF-ĸB, are dramatically increased in HeLa cells expressing Tax together with Urm1, as compared with cells expressing Tax alone. Quantitative RT-PCR, where the levels of Rantes (**a**) and IL-6 (**b**) mRNA are normalized against the housekeeping gene GAPDH, *P* < 0.001. **c** Comparison of the subcellular localization of interaction events between Urm1 and Tax, in relation to complex formation involving Tax and ubiquitin, versus Tax and SUMO-1, visualized using proximity ligation assay in HuT102 cells. **d** Treatment of HuT102 cells with arsenic/IFN counteracts the Urm1-depentent cytoplasmic shuttling of Tax, resulting in an increased accumulation of Tax1-Urm protein complexes in the nucleus, *n* = 42, *P* <  0.001

To investigate the functional impact of Tax urmylation on the ability of Tax to activate the NF-ĸB pathway, we assessed the effect of Urm1 addition on Tax-induced upregulation of transcription of NF-ĸB targets such as Rantes and interleukin-6 (IL6). Consistent with the increase of Tax-induced Diptericin transcription in *Drosophila*, we observed a significant elevation of Tax-induced transcripts for both Rantes (Fig. 3a) and IL-6 (Fig. 3b) in HeLa cells upon addition of Urm-1, supporting an important role for Urm1 in Tax-mediated cellular signaling.

Finally, we examined the interaction between endogenous Tax and endogenous Urm1 by proximity ligation assay in HuT-102 cells and compared it to other UBLs, which are known to modify Tax. Interestingly, whereas Tax - Urm1 complexes were predominantly localized in the cytoplasm, Tax - SUMO1 interaction was most frequently observed in the nucleus and no basal Tax-ubiquitin interaction was noted (Fig. 3c). We finally assessed how Tax-Urm1 complex formation was affected by arsenic/IFN treatment of HuT-102 cells, and discovered an increased nuclear targeting of urmylated Tax in response to treatment (Fig. 3d).

## Conclusions

We have uncovered a novel role of the UBL-molecule Urm1 as a modifier of the viral oncoprotein Tax, involved in the subcellular targeting and signaling activity of Tax. Specifically, we provide evidence that Urm1 is covalently conjugated to Tax in transfected HeLa cells and in HTLV-I transformed HuT-102 cells, as well as in *Drosophila* tissues. In all cases, Tax urmylation is associated with a size shift of Tax and clearly affects its subcellular localization by promoting a cytoplasmic accumulation of Tax. Most likely multiple Urm1 moieties attach to Tax via lysine residues 4-8, since neither of the tested mutants in this region were urmylated in our assay. Furthermore, Urm1-dependent cytoplasmic localization of Tax is correlated with NF-ĸB signaling, since the mRNA levels of *Rantes* and *IL*-*6*, both known as transcriptional targets of NF-ĸB, are dramatically increased in HeLa cells expressing Tax together with Urm1, as compared with cells expressing Tax alone. Interestingly, treatment of HuT-102 cells with arsenic/IFN increased the nuclear targeting of urmylated Tax. Our data put forward Tax as the first oncoprotein to be identified as a target of the UBL Urm1, and paves the way for further studies aimed at elucidating the outcome of Tax urmylation, such as pathogenicity and degradation mechanisms. In order to fully understand the functional implication of Tax urmylation, it will in addition be essential to investigate the crosstalk between the different UBLs that conjugate to Tax and experimentally address whether Tax ubiquitylation, SUMOylation and urmylation occur independently, sequentially, or simultaneously, in order to regulate Tax activation.

## Methods

### Fly stocks

Wild type *white*^*1118*^, *GMR*-*GAL4*, *Sgs3*-*GAL4* and *FB*-*GAL4* was from Bloomington *Drosophila* Stock Center, Indiana, USA. The RNAi line for Urm1 *P{GD15862}v48364* was from Vienna *Drosophila* Resource Center, Vienna, Austria [33]. *UAS:Urm1*^*WT*^ [32] and *UAS:Tax*^*WT*^ [34] have been described previously.

### Cells and Plasmids

HeLa cells were cultured and transfected with Lipofectamine 2000TM (Gibco, Invitrogen) as previously described [22]. PSG5 M-Tax lysine to arginine mutants Tax^K4-8R^, Tax^K1-3R^, Tax^K6R^, Tax^K6-7^, Tax^K7-8R^ and Tax^K1-10R^ have been previously described [18]. pcDNA4:Urm1 (DC01337) was from Abgent.

### Immunoprecipitation and Western Blot

HeLa cells or adult flies were lysed and sonicated in 2% SDS and 50 mM Tris-HCl, pH 8. Immunoprecipitation were performed in 50 mM Tris-HCl, pH 8, 200 mM NaCl, 0.1 mM EDTA, 0.5% NP-40, 10% glycerol, and protease inhibitors, with Tax antibodies (#168-A51 from the National Institutes of Health AIDS Research and Reference Reagent Program) and protein A-agarose (Sigma). Following washing and elution in sample buffer, immunoprecipitations and lysate controls were analyzed by Western blot, as previously described [8], using anti-myc(9E10) at 1:250 (Santa Cruz), anti-Tax at 1:250, anti-GAPDH at 1:20000 (Abnova), *Drosophila* anti-Urm1 at 1:500 [32] and anti-Tubulin at 1:5000 (Sigma). *Drosophila* immunoprecipitations were performed using lysates from fly heads expressing UAS:Myc-Tax and/or UAS:Flag-Urm1 under control of GMR-GAL4 (UAS/GAL4 system).

### In situ proximity ligation assays (Duolink) and confocal microscopy

HeLa or HuT-102 cells were fixed in methanol onto glass coverslips or by cytospin, respectively. Protein-protein interactions were visualized using the Duolink in situ proximity ligation assay (PLA) system (Olink Bioscience), employing anti-Tax, anti-Urm1 [32], anti-SUMO-1 C9H1 (Cell Signaling Technology), anti-ubiquitin FL-76 (Santa Cruz) and anti-Myc ab9106 (Abcam).

### Immunofluorescence

Immunofluorescence staining of *Drosophila* tissues was performed as previously described [32], employing the antibodies anti-Urm1 at 1:500 [32], anti-Myc (9E10) at 1:500 (Sigma), and DAPI for nuclear visualization. HeLa cells were stained with anti-Tax (as above) at 1:50, and anti-Myc ab9106 (Abcam).

### Quantitative PCR

Total RNA, extracted with TRIzol^®^ (Thermo Fischer Scientific), was template for cDNA synthesis with Random Hexamers and SuperScript^®^II Reverse Transcriptase (Thermo Fischer Scientific). Purification of DNA was performed using Clean and Concentrator™ kit (Zymo Research), followed by qPCR employing the KAPA SYBR FAST qPCR Master Mix (Kapa Biosystems). Primer sequences for Urm1 were 5′-GGGCGGAGTTACTATTTGGT-3′ and 5′-TCATAACCGATTTCACTCAAGTTT-3′ and for Diptericin 5′-GTTCACCATTGCCGTCGCCTTAC-3′ and 5′-CCCAAGTGCTGTCCATATCCTCC-3′.

IL-6 levels were assessed using the primers 5′-GGAGACTTGCCTGGTGAA-3′ and 5′-GCATTTGTGGTTGGGTCA-3′, whereas Rantes was monitored using 5′-ACCACACCCTGCTGCTTTGC-3′ and 5′-CCGAACCCATTTCTTCTCTGG-3′ primers.

## Abbreviations

ATL: adult T-cell leukemia/lymphoma
HTLV-1: human T-cell lymphotropic virus 1
HuT-102: human T-cell lymphoma cell line 102
IFN: interferon-alpha
NF-κB: nuclear factor kappa-light-chain-enhancer of activated B cells
SUMO: small ubiquitin modifier
UBL: ubiquitin like molecule
Urm1: ubiquitin-related modifier 1
